# Superovulation Does Not Alter Calcium Oscillations Following Fertilization

**DOI:** 10.3389/fcell.2021.762057

**Published:** 2021-11-04

**Authors:** Virginia Savy, Paula Stein, Min Shi, Carmen J. Williams

**Affiliations:** ^1^ Reproductive and Developmental Biology Laboratory, Durham, NC, United States; ^2^ Biostatistics and Computational Biology Branch, National Institute of Environmental Health Sciences, National Institutes of Health, Durham, NC, United States

**Keywords:** superovulation, oocyte, mouse, calcium oscillations, egg activation

## Abstract

Superovulation is a common approach to maximize the number of eggs available for either clinical assisted reproductive technologies or experimental animal studies. This procedure provides supraphysiological amounts of gonadotropins to promote continued growth and maturation of ovarian follicles that otherwise would undergo atresia. There is evidence in mice, cows, sheep, and humans that superovulation has a detrimental impact on the quality of the resulting ovulated eggs or embryos. Here we tested the hypothesis that eggs derived from superovulation have a reduced capacity to support calcium oscillations, which are a critical factor in the success of embryo development. Eggs were obtained from mice that were either naturally cycling or underwent a standard superovulation protocol. The eggs were either parthenogenetically activated using strontium or fertilized *in vitro* while undergoing monitoring of calcium oscillatory patterns. Following parthenogenetic activation, superovulated eggs had a slightly delayed onset and longer duration of the first calcium transient, but no differences in oscillation persistence, frequency, or total calcium signal. However, *in vitro* fertilized superovulated eggs had no differences in any of these measures of calcium oscillatory behavior relative to spontaneously ovulated eggs. These findings indicate that although subtle differences in calcium signaling can be detected following parthenogenetic activation, superovulation does not disrupt physiological calcium signaling at fertilization, supporting the use of this method for both clinical and experimental purposes.

## Introduction

Fertilization represents the union of two terminally differentiated gametes to form a single embryo capable of developing into a unique individual. Gamete fusion is only the beginning of the process of embryo development, but it sets in motion a series of events collectively termed “egg activation” that turn the unified gametes into a totipotent embryo capable of becoming a healthy individual. The key event of egg activation that triggers development in all species is a rise in the cytoplasmic calcium level (Kashir et al., 2013). What is unusual about mammalian eggs is that following the initial calcium rise, they go on to have a series of oscillations in cytoplasmic calcium levels that persist for several hours after fertilization. These calcium oscillations drive downstream events of egg activation including exocytosis of cortical granules, activation of calcium/calmodulin-dependent protein kinase II-gamma, resumption of the cell cycle, and pronucleus formation, all of which are essential for initiating proper embryo development (Stein et al., 2020). Persistent calcium oscillations can be triggered in the absence of sperm using the divalent cation strontium, resulting in parthenogenetic egg activation mediated by the transient receptor potential channel TRPV3 (Fraser, 1987; Carvacho et al., 2013). In the mouse, an inappropriate pattern of calcium oscillations following fertilization is associated with reductions in implantation efficiency, reduced development to term, and abnormalities in offspring growth (Ozil and Huneau, 2001; Ducibella et al., 2002; Ozil et al., 2005, 2006).

The pattern of calcium oscillations at fertilization is modulated by the amount of PLCζ released by the sperm but also by many factors intrinsic to the fertilized egg (Stein et al., 2020). Calcium oscillations depend on the amount of calcium in endoplasmic reticulum (ER) stores as well as egg factors that regulate how quickly calcium is released from the ER, cleared from the cytoplasm, and then pumped back into the ER such that sufficient calcium stores are available for the next calcium release event. Calcium homeostasis is regulated by the activities of calcium pumps and ion channels (Berridge et al., 2003). Sarco-endoplasmic reticulum calcium-ATPases pump calcium back into the ER, and plasma membrane calcium-ATPase pumps clear calcium from the cytoplasm by extruding it across the plasma membrane. These pumps depend on mitochondrial production of ATP. Calcium influx channels support calcium entry into the cytoplasm down a concentration gradient from the extracellular milieu; calcium influx is necessary for complete refilling of ER stores and continuation of calcium oscillations (Igusa and Miyazaki, 1983; Kline and Kline, 1992). Together, these activities allow the egg to tolerate the release of large amounts of calcium from the ER and to refill ER stores once the calcium release event has been completed.

All the molecular components needed to support calcium homeostasis, egg activation, and preimplantation embryo development, such as stored proteins, nucleic acids, and energy substrates, are generated during the oocyte growth phase. In a normal estrous cycle, ovarian follicles are recruited into the growing pool independent of gonadotropin hormones until the secondary follicle stage, when the oocyte is surrounded by several layers of granulosa cells (Strauss and Williams, 2017). Follicle stimulating hormone (FSH), secreted from the pituitary, initiates further follicle development to the antral stage, when fluid begins to collect between the granulosa cells. FSH and luteinizing hormone (LH) together promote continued survival and development of antral follicles toward the preovulatory stage (Chun et al., 1996; Sullivan et al., 1999). When FSH levels decline in response to estradiol-mediated negative feedback on the pituitary, antral follicles instead will undergo atresia. A select few “dominant” follicles continue to grow despite declining FSH levels. These follicles are either more sensitive to the available FSH or have developed sufficient responsiveness to LH to continue their developmental trajectory (Sullivan et al., 1999). As a result, a species-specific number of preovulatory follicles forms during each estrous cycle.

Successful superovulation was first reported almost 100 years ago, when whole pituitary tissue from donor animals was injected intramuscularly into rats and mice and resulted in either ovaries containing very large numbers of normal-appearing follicles or oviducts containing large numbers of ovulated eggs (Smith and Engle, 1927). Although the exact procedure has been modified extensively since that time, superovulation is commonly utilized to maximize the number of ovulated eggs available for either experimental purposes or for clinical use within assisted reproduction cycles. Superovulation works by providing high amounts of exogenous gonadotropin hormone so that non-dominant late secondary and early antral follicles, which are sensitive to gonadotropin stimulation and would otherwise be destined to undergo atresia, are instead maintained in the growing follicle pool and form preovulatory follicles. The preovulatory follicles can be allowed to ovulate spontaneously or induced to ovulate by administration of chorionic gonadotropin, which binds to the LH receptor and begins the ovulatory signaling cascade.

There is evidence in mice, cows, sheep, and humans that superovulation has a detrimental impact on the quality of the resulting ovulated eggs or embryos (Moor et al., 1985; Hyttel et al., 1986; Yun et al., 1989; Assey et al., 1994; Blondin et al., 1996; Van der Auwera, 2001; Baart et al., 2007; Fortier et al., 2008; Market-Velker et al., 2010; Lee et al., 2017). These findings could be explained by many factors, including accelerated oocyte growth, rescue of abnormal oocytes from atresia, and recruitment for ovulation of non-mature oocytes, all of which could lead to eggs that lack the maternally derived stores needed for developmental competence. Because calcium oscillatory patterns depend heavily on maternal components that regulate calcium homeostasis, we hypothesized that superovulation results in eggs that have abnormal patterns of calcium oscillations following parthenogenetic activation or fertilization. Small differences in calcium oscillatory behavior between spontaneously ovulated and superovulated eggs were observed following parthenogenetic activation, but there were no differences following *in vitro* fertilization (IVF). These findings indicate that calcium responses to sperm-induced egg activation are sufficiently robust to overcome subtle deficits in egg competence associated with superovulation.

## Methods

### Animals and Superovulation

C57BL/6J females (3–6 weeks old), C57BL/6J vasectomized males (2–8 months old), and B6SJLF1/J males (4–6 months old) were obtained from The Jackson Laboratory (Bar Harbor, ME).

Metaphase II-arrested (MII) eggs were collected from the oviducts of naturally cycling, 6-week-old females following overnight breeding to vasectomized males; vaginal plugs were checked to assure ovulation had occurred. For superovulation, 3-week-old females were primed by intraperitoneal injection of 5 IU of equine chorionic gonadotropin (Lee Biosolutions, Maryland Heights, MO) followed 46–48 h later by 5 IU human chorionic gonadotropin (Sigma Aldrich, St. Louis, MO). The superovulated females were also bred overnight to vasectomized males for consistency across both treatment groups; only vaginal plug-positive females were used.

All mice were sacrificed by CO_2_ asphyxiation and cervical dislocation. All procedures involving mice were conducted in accordance with National Institute of Environmental Health Sciences guidelines under approved animal care and use protocols.

### Parthenogenetic Activation, *in Vitro* Fertilization and Calcium Imaging

Eggs were collected at 9:00 am on the day the vaginal plug was detected, which corresponded to 14 h after human chorionic gonadotropin injection of superovulated females. Minimal Essential Medium with Hepes (Thermo Fisher, Waltham, MA) containing 0.1% PVA and 0.1% hyaluronidase (Sigma, St. Louis, MO) was used for egg collection and cumulus cell removal. In each biological replicate, eggs from 3–5 mice, either superovulated or naturally cycling, were pooled and treated with acidic Tyrode’s solution (pH 1.6) to remove the zona pellucida. The zona-free eggs were allowed to recover in KSOM medium (Millipore Sigma, Burlington, MA) for 30 min. The eggs were then loaded with the calcium indicator Fura-2 AM (5 μM; Thermo Fisher) for 30 min in KSOM containing 0.02% pluronic F-127 (Thermo Fisher) and then washed in fresh KSOM medium.

To monitor changes in cytosolic calcium, eggs obtained from superovulated or naturally cycling mice were adhered side by side to glass-bottom dishes (MatTek, Ashland, MA). This procedure ensured that the eggs in the two groups were cultured under exactly the same conditions. For parthenogenetic activation, eggs were adhered in 1.8 ml of Ca^2+^/Mg^2+^-free CZB medium (Gambini et al., 2020) without polyvinyl alcohol and, after calcium imaging was initiated, strontium (200 μl of 100 mM SrCl_2_ in Ca^2+^/Mg^2+^-free CZB) was added to achieve a final concentration of 10 mM. For IVF, eggs were adhered to Cell-Tak-treated (Thermo Fisher) dishes in 150 µl of BSA-free KSOM (Millipore Sigma). Forty-five µl of human tubal fluid medium (HTF; Millipore Sigma) containing 4 mg/ml BSA (HTF-BSA) were added to the drop to achieve a final concentration, once sperm were added, of 1 mg/ml BSA, and then the drop was covered with mineral oil.

Motile sperm were isolated from the epididymides of adult B6SJLF1/J males in HTF-BSA, via the swim-up method. Briefly, the epididymis and vas deferens were dissected and placed in 500 µl HTF-BSA covered with mineral oil. Under a dissection microscope, the tissue was cut several times and cultured for 15 min at 37°C to allow the motile sperm to swim out. The tissue was removed and 150 µl of swim-out sperm were carefully placed at the bottom of a clean tube containing 850 µl of HTF-BSA. The sperm were cultured for 60 min at 37°C to allow the motile sperm to swim up and capacitate. Sperm from the top layer of the culture medium were carefully transferred to a pre-warmed tube and the final sperm concentration was determined using a hemocytometer. Sperm were diluted in HTF-BSA to 10^6^ sperm/ml, then 5 µl were added to the IVF dish (195 µl volume) to achieve a final concentration of 25,000 sperm/ml.

Cytosolic calcium was measured by recording the intensity of fluorescence induced by 340 and 380 nm excitation and calculating the F340/F380 ratio. Ratio images were recorded every 7.5 s using a Hamamatsu ORCA-Flash4.0 LT + digital camera (Hamamatsu, Bridgewater, NJ) attached to a Nikon Ti inverted microscope with a Nikon S Fluor 20x/0.75 NA objective (Nikon Instruments, Melville, NY) and a Lambda 10-B Optical Filter Changer (Sutter Instruments, Novato, CA). Nikon NIS-Elements software was used for data acquisition and visualization. All experiments were performed at 37°C in a humidified atmosphere of 5% CO_2_, in an Okolab stage microenvironmental chamber enclosed in a microscope cage incubator (Okolab, Ambridge, PA).

For parthenogenetic activation, a total of four biological replicates and eight technical replicates were analyzed, including 78 eggs from superovulated females and 75 eggs from naturally cycling females. For IVF, a total of four biological replicates and eight technical replicates were analyzed, including 92 eggs from superovulated females and 68 eggs from naturally cycling females. Eggs of each group were either activated with strontium or fertilized and imaged at the same time, in the same medium drop, for each experimental replicate.

### Data Analysis and Statistical Tests

Significance of differences between the number of eggs obtained from naturally cycling and superovulated females was tested using the Mann–Whitney *U*-test, as the data were not normally distributed. The analysis was performed at significance level of 0.05 and CI of 95%, using Prism version 9.0.2.

A custom R function was developed for automated analysis of calcium imaging data and is available at https://www.niehs.nih.gov/research/atniehs/labs/assets/docs/q_z/savy_rscript.zip. The R-Script was used to determine the time to the first calcium transient, length of the first oscillation, area under the curve (AUC), number of oscillations for 60 and 120 min from the starting point of the first transient, number of oscillations per 10 min and persistence of oscillations for 110–120 min. Statistical tests were performed using GraphPad Prism. Because the data were not normally distributed, the time to the first transient, length of the first transient, AUC, and oscillation frequency were analyzed using a Mann–Whitney *U*-test. Data regarding oscillation persistence were analyzed using the Log-rank (Mantel-Cox) test.

## Results

We used the inbred mouse line, C57BL/6J, to test whether there were differences in calcium oscillatory patterns between eggs from naturally cycling (NC) and superovulated (SOV) mice. This choice was driven in large part because knockout facilities utilize this strain for the generation of the majority of their animal models. Furthermore, many transgenic and knockout lines are uniquely available on this background strain. As anticipated, far more eggs were obtained from SOV mice (median = 60.5 eggs) than from NC mice (median = 8 eggs; Mann–Whitney U = 41, n_NC_ = 20 n_SOV_ = 22, *p* < 0.0001; two-tailed).

**FIGURE 1:**
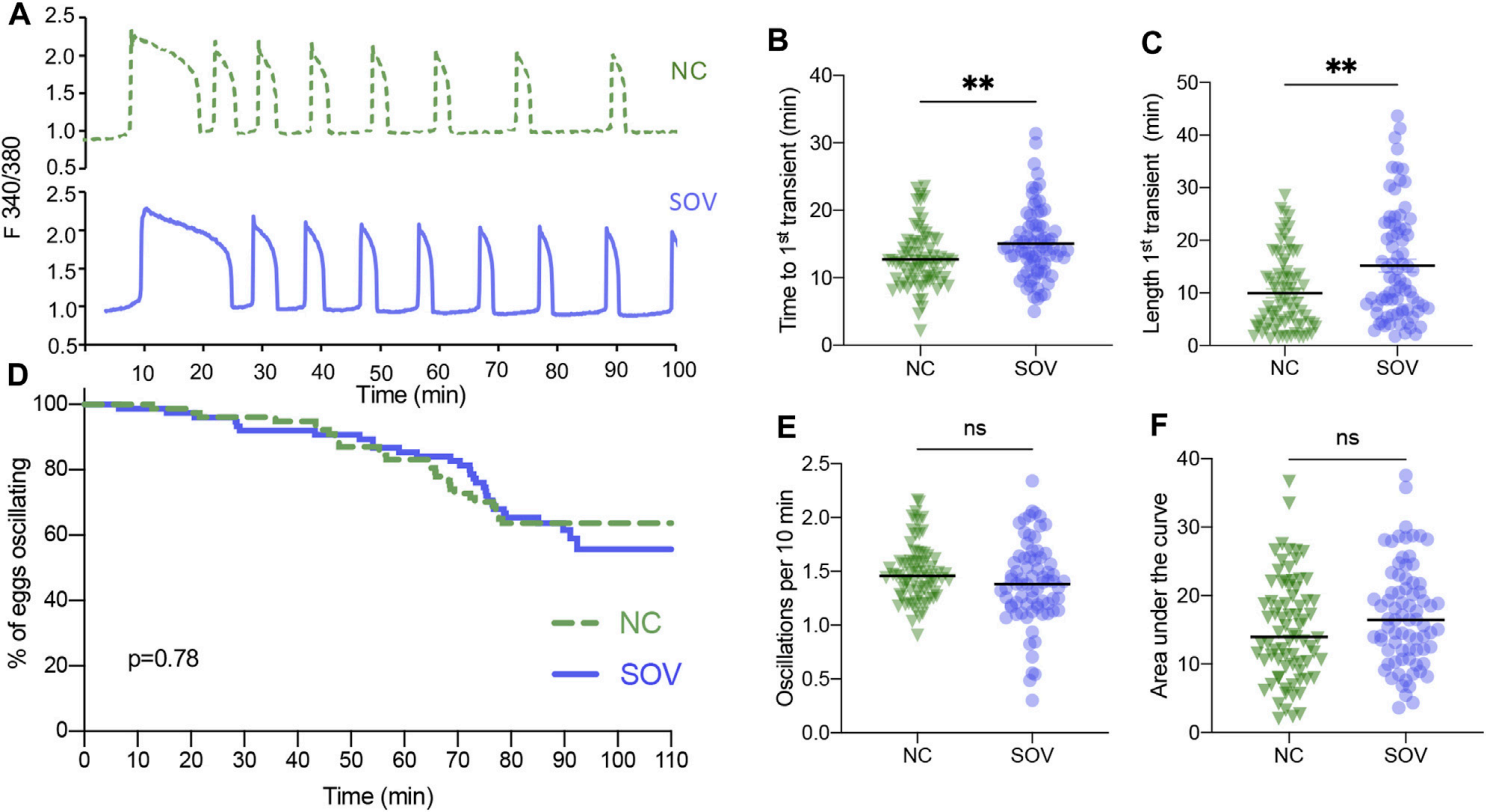
Calcium oscillatory patterns following strontium-induced parthenogenetic activation of MII eggs from naturally cycling (NC) and superovulated (SOV) mice. **(A)** Representative calcium traces. **(B)** Time to the first transient. **(C)** Length of the first calcium transient. **(D)** Percentage of eggs that continued to display calcium oscillations during 110 min. *p*-value indicated on graph. **(E)** Oscillation frequency averaged over the first 60 min of oscillations. **(F)** Area under the curve of calcium signal during the first 60 min following activation. Graphs in **(B,C,E,F)** show median and all individual data points. **p < 0.01; ns, no significant difference.

We first tested SOV and NC eggs for differences in calcium oscillatory patterns following strontium-induced parthenogenetic activation, which prevents any potential variability that could be introduced by differences in individual sperm during IVF. SOV eggs took slightly longer to begin oscillating and the first transient was about 50% longer (Figures 1A–C), indicating that there were differences in strontium sensitivity and in cytoplasmic calcium handling in the presence of strontium. A detailed analysis of additional parameters including the percentage of eggs that continued to display calcium oscillations (oscillation persistence), oscillation frequency, and the area under the curve of calcium signal revealed no significant differences between the two groups (Figures 1D–F).

**FIGURE 2:**
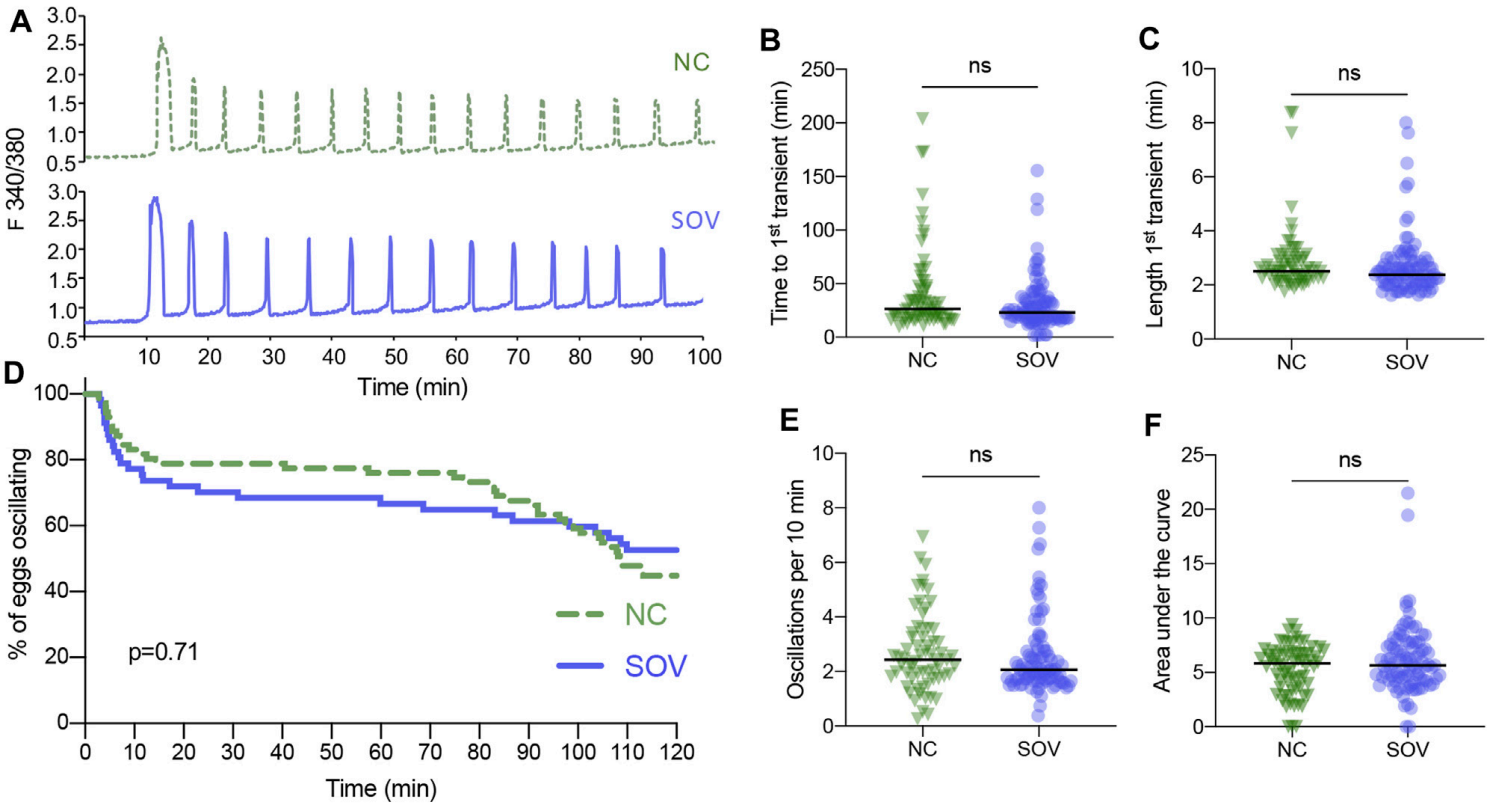
Calcium oscillatory patterns following fertilization of MII eggs from naturally cycling (NC) and superovulated (SOV) mice. **(A)** Representative calcium traces. **(B)** Time to the first transient. **(C)** Length of the first calcium transient. **(D)** Percentage of eggs that continued to display calcium oscillations during 120 min. *p*-value indicated on graph. **(E)** Oscillation frequency averaged over the first 60 min of oscillations. **(F)** Area under the curve of calcium signal during the first 60 min following fertilization. Graphs in **(B,C,E,F)** show median and all individual data points. ns, no significant difference.

A similar analysis was performed of the calcium oscillatory responses of SOV and NC eggs to sperm-induced egg activation. There were no obvious differences in the patterns of calcium oscillations, the time until the first transient, or the duration of the first transient (Figures 2A–C). Likewise, there were no significant differences in calcium oscillation persistence, frequency, or total calcium signal (Figures 2D–F). These findings suggest that superovulation does not impact fertilization-induced calcium oscillatory patterns to any measurable degree.

## Discussion

Here we tested in a highly controlled fashion whether superovulation causes abnormalities in calcium signaling following strontium-induced parthenogenetic activation or IVF in the mouse. We found that strontium-induced oscillations were slightly different in superovulated eggs, with a small delay in initiation and longer total duration of the first transient. Because TRPV3 mediates strontium-induced egg activation, differences in the amount of TRPV3 on the egg surface could explain the altered time to the first transient (Carvacho et al., 2013). In addition, differences in the ability of the egg to clear large amounts of calcium from the cytoplasmic compartment in the presence of strontium could explain the prolonged first transient. Our findings are similar to previous observations that eggs from aged mice differ in some of their calcium oscillatory responses to strontium relative to those in eggs from young mice, whereas these differences are not observed following more physiological egg activation methods (microinjection of either sperm heads or cRNA encoding the PLCζ protein) (Haverfield et al., 2016).

Despite our prediction to the contrary, the data clearly showed that in a commonly utilized inbred strain, C57BL/6J, superovulation according to a standard protocol does not alter the calcium oscillatory patterns at fertilization. It is possible that alternate superovulation protocols or repetitive superovulation could produce a different result. Superovulation maximizes the numbers of ovulated eggs that can be obtained for study from each mouse, reducing the numbers of animals that must be euthanized for experimental purposes by about 10-fold based on our data. Therefore, this technique is routinely performed to obtain oocytes and early embryos in laboratories and animal facilities. Our findings demonstrating normal fertilization-induced calcium oscillatory patterns despite superovulation is reassuring regarding interpretation of experiments examining factors that influence calcium signals given that these types of experiments are almost always performed with eggs obtained by superovulation. Our data support the use of superovulation in future studies of calcium signaling at fertilization, but suggest caution in the interpretation of results obtained in experiments utilizing chemical methods to activate superovulated eggs.

Despite being a widespread practice, superovulation is associated with abnormalities in several phenotypic features in oocytes and ovulated eggs. For example, an increased incidence of meiosis arrest prior to metaphase II, asynchrony between nuclear and cytoplasmic maturation, abnormalities in subcellular structure, and abnormal patterns of protein synthesis have all been observed in various mammalian species following superovulation (Moor et al., 1985; Callesen et al., 1986; Hyttel et al., 1986; Yun et al., 1989; Assey et al., 1994). Given that the hormone injections only rescue late secondary and antral follicles, these differences are clearly a consequence of inadequate preparation for oocyte maturation during later stages of follicle development. Although the oocyte is transcriptionally inactive after reaching the antral stage, translation continues to occur, creating additional maternal components to support maturation, and embryo development. In addition, mRNAs and long noncoding RNAs are transferred to the oocyte from the surrounding cumulus cells, at least some of which are translated and may contribute to oocyte competence to undergo maturation (Macaulay et al., 2014, 2016). Superovulation could accelerate late follicle development enough that there is insufficient time for the accumulation of specific maternal components that could explain the altered sensitivity to strontium in SOV and NC eggs. However, the absence of differences in fertilization-induced calcium dynamics suggests that adequate amounts of the maternal components required for physiological calcium homeostasis are already present prior to superovulation or, alternatively, that these components are not affected in a detrimental fashion by the superovulation protocol.

Unlike oocyte maturation defects, which occur prior to fertilization, some superovulation-associated abnormalities are observed following fertilization. Reduced competence to develop to or beyond the 16-cell stage, both *in vivo* and *in vitro*, abnormalities in acquisition and maintenance of methylation marks on imprinted genes and fetal growth retardation all are associated with superovulation protocols in cows and/or mice (Blondin et al., 1996; Van der Auwera, 2001; Fortier et al., 2008; Market-Velker et al., 2010). Differences in embryo development likely result from a lack of accumulation of other maternal components in the final period of follicle growth to the periovulatory stage that are not related to the calcium signaling toolkit. Future studies will be needed to determine the identity of such components or to develop superovulation protocols that do not disrupt preovulatory follicle development.

## Data Availability

The raw data supporting the conclusion of this article will be made available by the authors, without undue reservation.
